# HIV Vaccine Mystery and Viral Shell Disorder

**DOI:** 10.3390/biom9050178

**Published:** 2019-05-08

**Authors:** Gerard Kian-Meng Goh, A. Keith Dunker, James A. Foster, Vladimir N. Uversky

**Affiliations:** 1 Goh’s BioComputing, Singapore 548957, Singapore; 2 Center for Computational Biology, Indiana and Bioinformatics, Indiana University School of Medicine, Indianapolis, IN 46202, USA; kedunker@iupui.edu; 3 Department of Biological Sciences, University of Idaho, Moscow, ID 83844, USA; foster@uidaho.edu; 4 Institute for Bioinformatics and Evolutionary Studies, University of Idaho, Moscow, ID 83844, USA; 5 Department of Molecular Medicine, Morsani College of Medicine, University of South Florida, Tampa, FL 33612, USA; vuversky@health.usf.edu; 6 Institute for Biological Instrumentation, Russian Academy of Sciences, Moscow Region, Pushchino 142290, Russia

**Keywords:** HIV, intrinsic disorder, unstructured, immune escape, glycoconjugate, smallpox, polio, rabies, yellow fever, herpes, hepatitis

## Abstract

Hundreds of billions of dollars have been spent for over three decades in the search for an effective human immunodeficiency virus (HIV) vaccine with no success. There are also at least two other sexually transmitted viruses, for which no vaccine is available, the herpes simplex virus (HSV) and the hepatitis C virus (HCV). Traditional textbook explanatory paradigm of rapid mutation of retroviruses cannot adequately address the unavailability of vaccine for many sexually transmissible viruses, since HSV and HCV are DNA and non-retroviral RNA viruses, respectively, whereas effective vaccine for the horsefly-transmitted retroviral cousin of HIV, equine infectious anemia virus (EIAV), was found in 1973. We reported earlier the highly disordered nature of proteins in outer shells of the HIV, HCV, and HSV. Such levels of disorder are completely absent among the classical viruses, such as smallpox, rabies, yellow fever, and polio viruses, for which efficient vaccines were discovered. This review analyzes the physiology and shell disorder of the various related and non-related viruses to argue that EIAV and the classical viruses need harder shells to survive during harsher conditions of non-sexual transmissions, thus making them vulnerable to antibody detection and neutralization. In contrast, the outer shell of the HIV-1 (with its preferential sexual transmission) is highly disordered, thereby allowing large scale motions of its surface glycoproteins and making it difficult for antibodies to bind to them. The theoretical underpinning of this concept is retrospectively traced to a classical 1920s experiment by the legendary scientist, Oswald Avery. This concept of viral shapeshifting has implications for improved treatment of cancer and infections via immune evasion.

## 1. Introduction

### 1.1. The Mystery of the Elusive HIV Vaccine

The search for an effective human immunodeficiency virus (HIV) vaccine has spanned over 30 years with hundreds of billions of dollars spent with no success [1]. The repeated standard textbook explanation for this mystery is that because of the retroviral nature of HIV, its replication involves an error-prone reverse RNA to DNA transcription, making HIV vulnerable to mutation and thereby providing more means for escaping the host immune system [2,3,4,5]. While there is likely some truth to this, a closer look at all available data reveals that such an explanation is limited as there are several counterexamples for this hypothesis. For instance, if this would be the case for all retroviruses that utilize the error-prone reverse transcription as the definition of retrovirus entails, why then effective vaccines have been found for HIV’s horse cousin, equine infectious anemia virus (EIAV), at least since 1973? [6,7,8,9,10]. Furthermore, it should be noted that HIV is not the only virus, for which an effective vaccine is yet to be found despite enormous efforts. The hepatitis C virus (HCV) and herpes simplex virus-2 (HSV-2) are such examples, and, incidentally, these are not retroviruses, which seem to contradict the aforementioned textbook dogma. In fact, HCV and HSV-2 are single-stranded RNA and double-stranded linear DNA viruses, respectively. It would seem strangely coincidental that HIV, HCV, and HSV-2 all have strong sexual transmission components. The connection between sexual transmission and the unavailability of an effective vaccine is something that has never been and simply cannot be explained by the current textbook paradigm.

Given the serious deficiencies of the current textbook paradigm, it is clear that a mystery has been quietly plaguing the biomedical community for over three decades, and a new explanatory framework that could adequately address the aforementioned puzzles is much needed. This paper describes the concept of viral shell disorder or shapeshifting that was first introduced by us in a 2008 study that reported a strange characteristic not previously noticed in the outer shell and matrix of the HIV-1, namely the HIV-1 matrix was found to be highly disordered [11]. In fact, using advanced computational techniques (e.g., PONDR^®^-VLXT) we showed that depending on a strain, the percentage of intrinsic disorder (PID) in HIV-1 matrix protein (p17) can be as high as 70%, the levels, which are very rare in the outer shells of other viruses [11,12]. A decade has passed since the publication of that paper, and plenty of both computational and experimental data have been made available. For instance, we now know that the basis of its theoretical framework can be found in the 1920s classical experiments of Oswald T. Avery and Walther F. Goebel, who showed that bacterial polysaccharides are essentially ineffective as vaccines, but may become efficient being held together as a rigid conjugate with proteins [3,12,13,14,15]. Furthermore, a shell disorder database of over 300 viruses and strains has been built with much of its data made publicly available [3,16]. With the availability of information for a wide variety of viruses that include polio, rabies, HCV, HSV, and yellow fever viruses (YFV) [3,11,12,17,18,19,20,21,22,23,24,25,26] important comparisons become feasible as we shall see below.

### 1.2. Protein Intrinsic Disorder

While structural biology tells us that structures are responsible for many functions of proteins, it has also been long known that there are many proteins or protein regions that do not have unique structures, but yet have important functions [27,28,29,30]. Having known which proteins (or protein regions) are easily crystallizable and which are not, a neural network can be trained on the sequences of the appropriate proteins to give rise to a disorder predictor. The aforementioned peculiarity of HIV was discovered using one of such disorder predictors, PONDR^®^-VLXT [11]. Historically, PONDR^®^-VLXT was the first intrinsic disorder predictor, and it uses a neural network that takes a protein sequence as input and provides disorder or order score for each residue as output (http://www.pondr.com) [31,32]. PONDR^®^-VLXT was chosen as the ideal predictor for our studies, as it is known to be a sensitive tool for predicting disorder-based protein-protein interactions [33,34,35]. This predictor has been shown to be a reliable tool in providing insights into a long list of medically important viruses, such as the 1918 H1N1 influenza virus, Ebola, Middle Eastern Respiratory Syndrome, coronavirus (MERS-CoV), Severe Acute Respiratory Syndrome coronavirus (SARS-CoV), and *Flaviviridae* viruses [3,11,17,18,19,20,22,25,26]**.**

### 1.3. Viral Shells 

**Figure 1:**
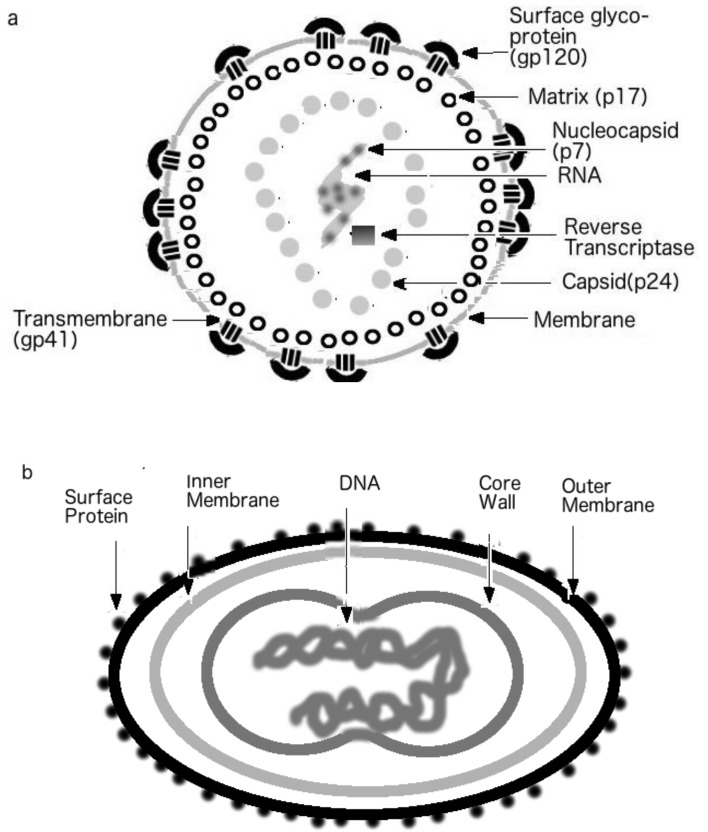

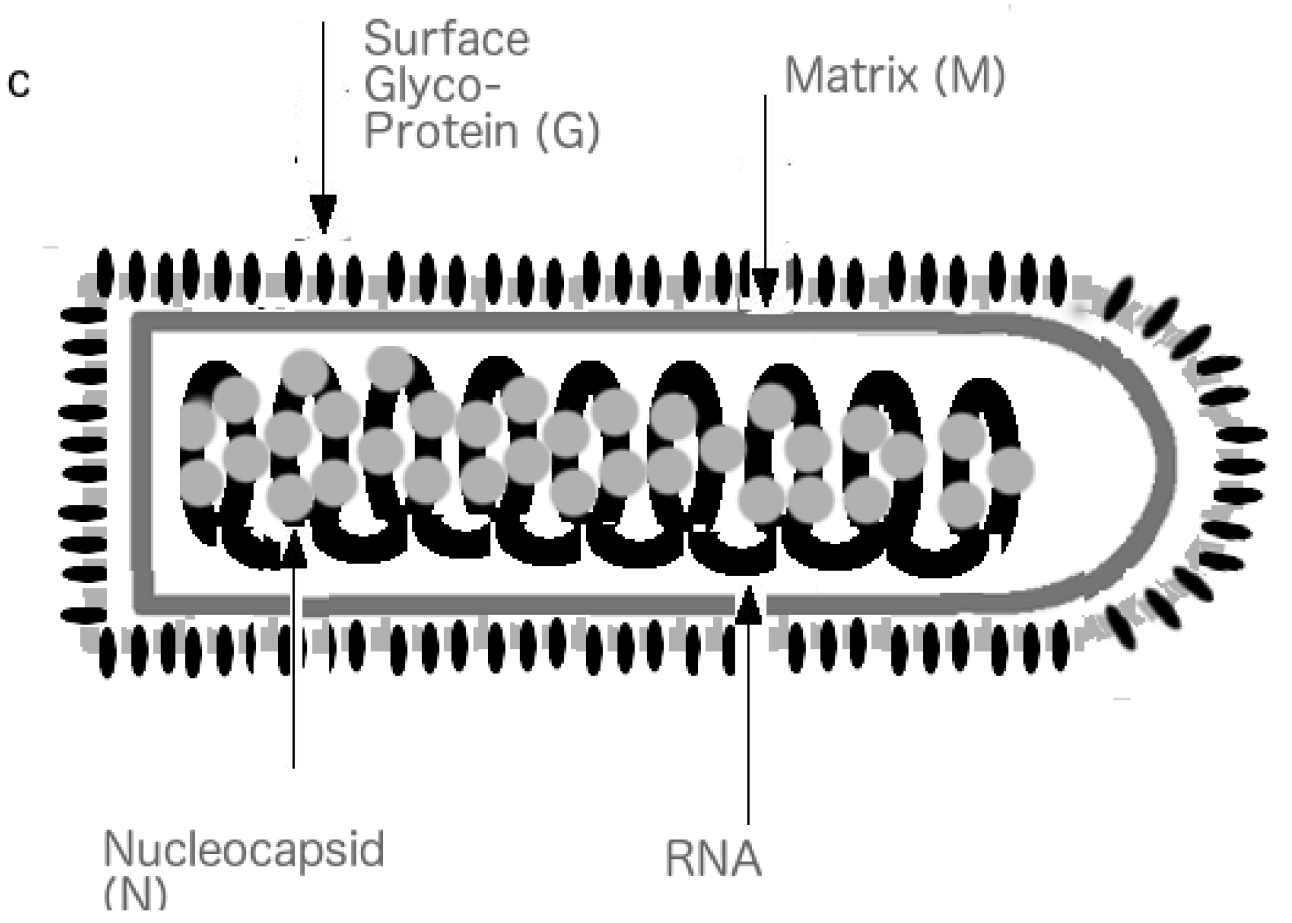
The shells of various viruses. (**a**) Virion of human immunodeficiency virus (HIV). (**b**) Rabies virus. (**c**) Variola (Smallpox) virus. (Figures reproduced with the permission of Gerard KM Goh, 2017).

Virion or virus body includes an RNA or DNA genome surrounded by a protective, virus-coded protein coat. These protective capsids are formed as single or double protein shells and consist of only one or a few structural protein species. An outermost layer of capsid consists of a glycoprotein and can be followed by at least one more protein shell layer. Some viruses also contain an envelope, which is an additional covering usually derived from the modified host cell membranes [36,37]. The number of shell layers depends on the type of virus. Figure 1 shows examples of shells of various viruses. The HIV and other retroviruses have three layers, the matrix, the capsid, and the nucleocapsid, as seen in Figure 1a. The rabies virus, on the other hand, has only two layers (Figure 1b). Interestingly, Figure 1c shows that smallpox virus has multiple shell layers. Since the number of shell layers might define the sustainability of viruses, it is not surprising that smallpox can survive in the environment for a long time, a characteristic known to the laboratory workers who handle the virus. In fact, a 19th century English medical journal (Medical and Physical Journal No. 199) described, based on a previous report [38], an incident in England, where a worker clearing a 30-year-old grave was infected with smallpox [3,39,40]. 

A question that comes into our mind is whether the hardness of the shell (and not only its complex multi-layer structure) supports this observation. We shall see that the use of disorder predictors (e.g., PONDR^®^ VLXT) represents a means to answer this question and to gain important knowledge about the shells of variola and other important viruses. 

It should be noted also that viruses come in different shapes and complexities. Viruses can be round and ball-shaped, as in case of HIV-1 (Figure 1a) or be bullet-shaped as seen in rhabdoviruses that include the rabies virus (Figure 1b), or have the dumbbell shape as found among poxviruses, such as variola virus (Figure 1c). Different viruses also show various levels of their proteome complexity. For example, the HIV genome encodes only 15 proteins, with repeating units of one protein for each shell level. The herpes simplex virus (HSV), on the other hand, has as many as 84 proteins, with its tegument (matrix, outer shell) containing as many as 20 different proteins [2,41]. Another complex DNA virus is the smallpox virus that contains over 200 proteins [2].

## 2. Virus Selection: Classical Vaccine Successes vs. HIV, HSV-2 and HCV

For this study, a set of viruses was carefully selected as seen in Table 1. They were chosen as representatives of viruses for which the effective vaccines are available or not. HIV, HSV-2, and HCV are good references for viruses that have no available vaccines as of yet [3,26]. HIV was first discovered in 1984, and efforts for vaccine development began almost immediately, whereas HSV-2 has been known since ancient times and the first vaccine effort began in the 1920s [3]. In 2009, a very large HIV vaccine trial took place in Thailand that failed disastrously. For HSV, a large vaccine trial involving 8323 women took place in 2012 and, like the HIV trial, it ended in failure especially for HSV-2 [41]. By contrast, investigational vaccine was effective in preventing HSV-1 genital disease and infection [41]. Interestingly, HSV-2 is usually spread via sexual intercourse, whereas HSV-1 can be spread by kissing, even though the two viruses are closely related [42]. For this reason, HSV-2 is highlighted in our selection of viruses. Importantly, that all three viruses, HSV-2, HCV, and HIV, have a sexual component as a major transmission mechanism. It should be noted that these three viruses were not deliberately chosen for their sexual transmission, but, rather, for their current lack of an effective vaccine.

Discovered in 1988, HCV has been known that, similar to HIV, HCV is primarily spread through illicit drug use, it has been originally observed that HCV is not easily transmitted via sexual intercourse. However, more recent data indicated that HCV can be easily sexually transmitted, when at least one partner is HIV positive [43,44,45]. Furthermore, it is also more easily transmitted via anal sex [43]. HCV belongs to the genus Hepacivirus of the Flaviviridae family [26], which makes it a somewhat distant relative to the mosquito-borne YFV and dengue viruses that belong to the genus Flavivirus, and, thus, making HCV and YFV an interesting comparative pair. HCV is physiologically different from YFV, since it has no membrane protein layer, thereby making its core proteins the outermost shell layer. 

The historically important viruses were chosen among viruses with established efficient vaccines. They are smallpox, rabies, polio, and yellow fever viruses. EIAV, which is a horse cousin of HIV, has also been selected. Because it has the same virion physiology as HIV, its inclusion provides means for a comparative study. Although for ages, smallpox was a deadly curse killing a third of those infected with variola virus, this disease is now eradicated, and an effective vaccine using cowpox was discovered by Edward Jenner in 1796 [4,46,47]. An effective rabies vaccine was discovered by the great Louis Pasteur in the dawn of modern microbiology in the late 19th century [48]. Poliovirus, which spreads mainly by fecal-oral routes among humans, is also an ancient threat, for which effective vaccines developed by Jonas Salk became available in the 1950s [49]. YFV is a highly pathogenic virus spread by mosquitoes, for which an effective vaccine was first discovered by Max Theiler in 1937 [50].

## 3. Protein Intrinsic Disorder of Viral Shells: Abnormality of the HIV Outer Shell

Table 1 introduces shell proteins analyzed in this study. It is easy to analyze “simpler” viruses, such as HIV, as its shell contains only repeating units of p17, p24, and p7 in the matrix, capsid, and nucleocapsid, respectively (see also Figure 1a) [2,11]. However, there are more complex viruses, such as variola and herpes simplex viruses, which have multiple proteins at each shell layer. To overcome this complexity that could involve analysis of over 20 proteins per layer for each complex virus, only major shell proteins are considered in such viruses as seen in Table 1. Although different viruses typically have multiple layers in their shells, the poliovirus has a unique feature shared with its picornavirus relatives, namely, it possesses one-layered shell, the capsid that is made up of multiple proteins [19]. In the case of poliovirus, there are four proteins, VP1-4, that form a complex in the shell [2,3], and, therefore, these four proteins, VP1-4, were analyzed in this study.

Table 2 represents the percentages of intrinsic disorder (PID) in the shell proteins of various viruses and shows that the outer shell (matrix) of HIV-1 is characterized by a remarkably high level of intrinsic disorder. In fact, the HIV-1 matrix means PID of 56.5 ± 10.8 is the highest PID among all outer shell proteins, with the only exception being a HSV-2 tegument (matrix) protein. 

**Figure 2:**
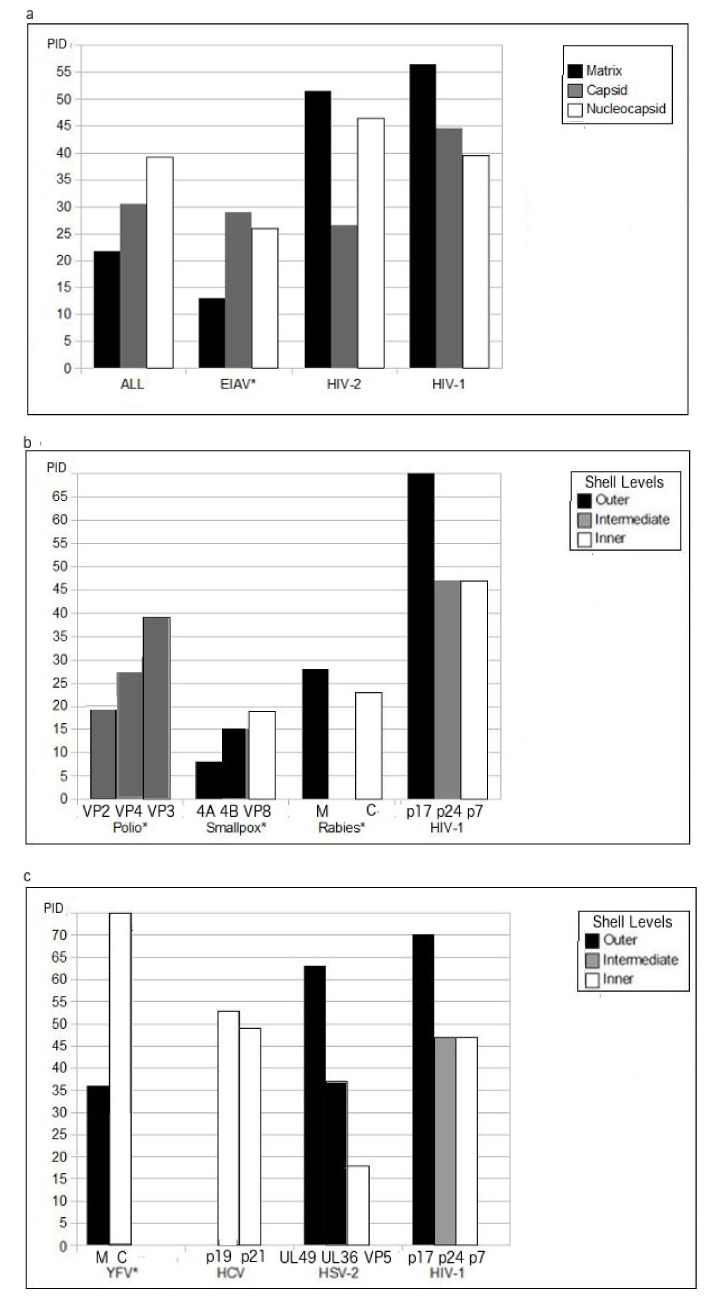
Comparison of PID of shell proteins of related. (**a**) Retroviruses: HIV vs. EIAV (**b**) HIV-1 vs. polio, smallpox and rabies viruses. (**c**) YFV vs. HCV, HSV and HIV-1. (*) denotes viruses for which effective vaccines are available. For illustrative purposes, only the tegument proteins with the highest and lowest PIDs are shown. Figure 2a shows the mean PID, whereas, in (**b**,**c**), each PID represents maximal PID found for the specific protein. The reason for the use of both mean and maximal values is that the mean PID alone can be quite misleading for the readers, and sometimes maximal number paints a more accurate picture. “ALL” refers to the mean shell PIDs of viruses in general as seen in the current database of over 300 viruses and strains. YFV M (Matrix) and C (Capsid) PIDs are displayed.

However, the corresponding HSV protein with the highest PID is one of the major tegument proteins in this complex virus. A clearer picture of the oddity of the HIV matrix protein can be seen in Figure 2a, where the mean shell PIDs of viruses in general (“All”, derived from a shell disorder database of over 300 animal viruses and strains [11,16] are compared to those of HIV-1 and HIV-2. It is important to note that the pattern of shell PIDs of viruses in general is completely different from that of HIV. More specifically, instead of a stepwise increase in PIDs as one move towards the innermost layer of the shell in the case of viruses in general, the HIV behavior is diametrically opposite. The reason for the ascending disorder levels in proteins of different shell layers for most viruses is that the outer shells play larger roles in protecting the virion from damage caused by physiological and non-physiological environments. However, HIV and other sexually transmitted viruses are not exposed to the harsh environments, and, therefore, have the luxury of using its outer shell to evade the host immune system. 

### 3.1. The HIV vs. EIAV Vaccine Puzzle: EIAV’s Hard Shells

**Figure 3:**
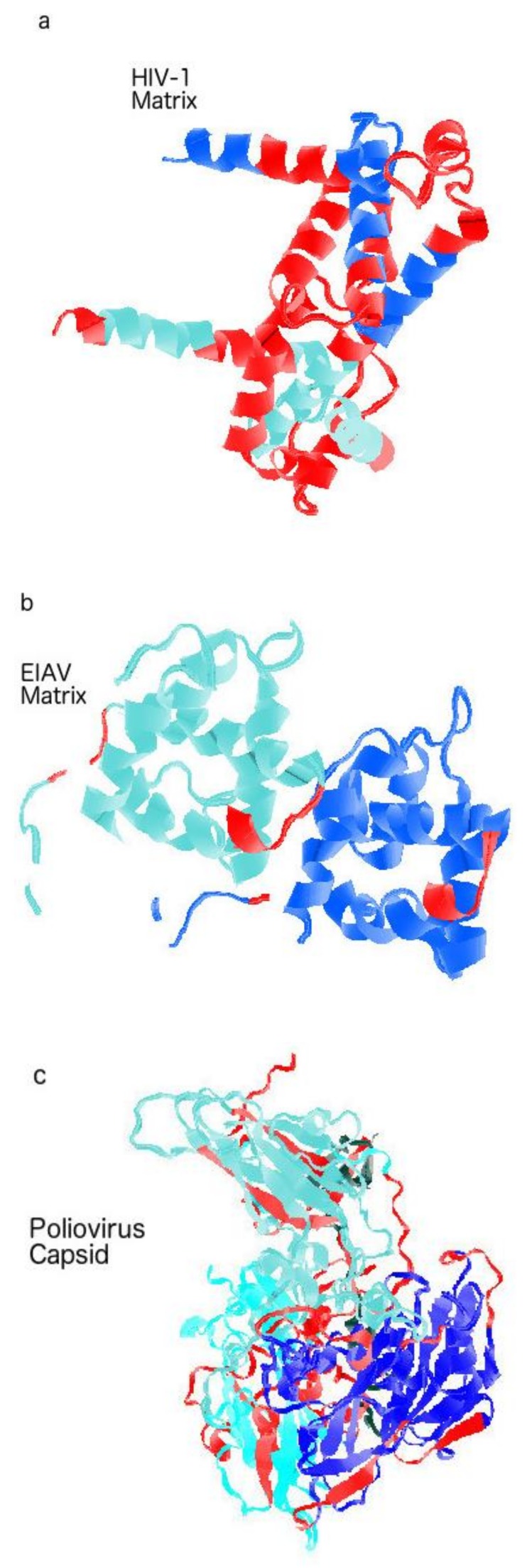
Crystal Structures with Disorder Annotated in Red. (**a**) A highly disordered HIV matrix (1hiw.pdb). (**b**) A highly ordered EIAV matrix (1hek.pdb). (**c**) Complex of poliovirus capsid proteins (1p02.pdb). The varying non-red colors in each sample denote different chains. The Protein Data Bank (PDB) structures without disorder annotations are available at: http://www.ncbi.nlm.nih.gov/structure/. A JAVA program was written to generate codes that could be read by Jmol. The JAVA program reads the disorder information from the MYSQL shell disorder database.

Similar to HIV, EIAV is a lentivirus. It infects horses via blood-sucking insects. EIAV has a case-fatality ratio (CFR) between 30–70% and could devastate horse ranches. Effective vaccines have been found since 1973 or earlier [8,9,10,36,51]. Some of the vaccines are known to have as high as 100% efficacy. In contrast, hundreds of billions of dollars have been spent for over 30 years in search of a HIV vaccine without any success. What is the mystery here? The answer can be found in Table 2 and Figure 2b, which show that all three layers of the EIAV shell are well-ordered (Matrix PID: 13 ± 0.1%; Capsid PID: 29 ± 0.1%; Nucleocapsid PID: 26 ± 0.1%) [11,25]. The HIV, on the other hand, has a high matrix disorder, but moderate levels of disorder for the inner shell layers. A sharp contrast can be seen in the X-ray crystal structures presented in Figure 3, where HIV-1 matrix protein has long stretches of disorder as represented by red color (Figure 3a) but only few disordered regions can be found in the EIAV matrix (Figure 3b). The found differences in disorder levels can be explained in terms of viral evolution and modes of transmission. The EIAV finds greater fitness for well-ordered and rigid virus particles that are able to survive in the mouthpiece of the insect, given that they will be exposed to harsh environment, such as saliva, containing proteolytic enzymes during its transportation between horses. For this reason, the EIAV cannot “afford” a soft disordered matrix that would help this virus to evade the host immune system, as seen for the sexually transmitted HIV. 

### 3.2. Outer Shell Hardness and the Classical Vaccine Successes: Smallpox, Rabies, Polio and YFV

Table 1, Table 2 and Figure 2 show that the classical viruses, for which effective vaccines were successfully developed, all are characterized by the remarkably low PIDs for the outer shell layers. For instance, all major shell proteins of variola virus are highly ordered, having PID scores below 30% [3]. The fact that both inner and outer shells are highly ordered corroborates with the fact that the smallpox virus has been historically known to persist in the environment for a long time. On the other hand, the fact that its major shell proteins are ordered could explain the reason that the smallpox vaccine was the earliest vaccine found. The rabies virus illustrates the same case but not to such extremes. The rabies virus has matrix and nucleocapsid PIDs below 30% [11], which are considered low. This hardness of the shell can explain the fact that the rabies vaccine is one of the first to be discovered during the dawn of modern microbiology.

The poliovirus is a bit more enigmatic. It has only one shell layer, the capsid, that has a complex of four proteins, VP1-4 [2,3,19]. The complexation of proteins allows poliovirus and its *Picornaviridae* cousins to have large fecal-oral transmission components through a formation of a rigid capsid as seen in Figure 3c. Furthermore, disorder helps with the complexation since disorder plays a role in recognition of binding partners. Nevertheless, when we measured the PID for each of the proteins, telltale signs of relative order necessary for its fecal-oral transmission is seen. We can see that the highest PID among the polio capsid proteins is still below 40%, while the lowest PID is less than 20%. These figures are still considered low in comparison to the HIV matrix PID that could reach a high of 70% (see Figure 2b). 

### 3.3. Influenza Virus: No Highly Disordered Outer Shell Like HIV, HSV and HCV

The standard textbook paradigm, used to explain the HIV vaccine mystery, is sometimes compared to the difficulty of finding an effective influenza vaccine as an analogous example of vaccine failure as the result of rapid mutations [4]. A counter argument for such analogy is that, unlike HIV, there are vaccines for influenza available. Their effectiveness varies each season depending on the successful anticipation and matching of oncoming strains for the season. According to the Center for Disease Control (CDC), influenza vaccine can reduce the chances of serious illness by 40–60% (https://www.cdc.gov/flu/about/qa/vaccineeffect.htm). The matrix PID mean (35.2 ± 1.6) for influenza virus as seen in Table 1**,** looks nothing like those of HIV, HSV or HCV and thus seems to lend support towards the idea of greater ease and feasibility of finding effective vaccines for influenza in contrast to HIV. We therefore argue that problems in the development of a successful HIV vaccine are fundamentally different from those of influenza.

## 4. Viral Shapeshifters of Another Kind: Trojan Horse with Inner Shell Disorder 

The YFV shell disorder pattern is telling us something not seen in the rest of the viruses. While its outer shell PID is close to 35%, the PID of its inner shell protein jumps to above a whopping 70% [12,25]. It is the only virus in our sample that has a moderately ordered outer shell but highly disordered inner shell. As mentioned, an effective vaccine against the YFV infection was discovered in 1938, which again re-affirms the paradigm of ordered outer shell and vaccine success. The highly disordered inner shell is something that has been seen not just in the YFV, but also in other highly pathogenic viruses, such as SARS-CoV and Ebola virus [3,11,12,20,22,25,26], even though the case of rabies tells us that a highly disordered inner shell is not an absolute requirement for virulence. While a highly disordered outer shell is a characteristic of a virus that can be described as a true viral “shapeshifter” that uses structural flexibility of their outer shells to directly evade the host immune system, a highly disordered inner shell found in YFV, SARS-CoV, and Ebola suggests the existence of a viral shapeshifter of another kind, a kind of “trojan” horse. More specifically, the disordered inner shell may help the virus to multiply rapidly before the immune system can respond. It does so through the protein promiscuity arising from the greater levels of protein disorder. In other words, in these cases, the virulence arises from the ability of the virus to multiply rapidly especially in vital organs [25,26]. The inner shell proteins (and sometimes also the outer shell proteins) usually play vital roles in the replication of the virus by attaching themselves to the proteins of the host cell [52]. By being disordered and thus promiscuous, the viral protein is able to bind to a greater variety of proteins that it would not normally bind to [11,12,25,26,30,42,53,54].

## 5. HIV, HSV-2 and HCV: Presence of Disordered Proteins at the Outer Shell and the Lack of Effective Vaccines

While the major core protein of HSV-2 is essentially ordered, the major tegument (matrix) shows a mix in levels of disorder [3]. A closer inspection reveals that the HSV tegument complex PID has some resemblance to that of HIV, unlike the poliovirus. Table 1 lists the major tegument proteins and their respective PIDs [3], whereas Figure 2c shows the lowest and the highest tegument PIDs found for HSV-2 and reveals that PID of one protein is over 60% and PID of another protein is around 50%, with no proteins with PIDs below 30%. 

The YFV shell PIDs are shown in Figure 2C. Since YFV is related to HCV, an interesting comparison can then be made, as HCV lacks a membrane layer, which forces HCV core protein to take the positions of the membrane proteins found in enveloped viruses [2,26]. With this distinction in mind, the HCV core PID is about 50%, in contrast to the PID of 35% found for the YFV membrane protein [26]. Therefore, HCV has some resemblance to HIVs, but not to the extreme seen in HIV-1 as shown in Figure 2c.

### 5.1. HSV, HCV and HIV: Similarities and Differences in Shell Disorder and Transmission Evolution

While we have seen the stark similarities in the shell disorder of HCV, HSV and HIV, we also need to keep in mind the somewhat subtle differences among them in terms of transmission evolution and genetics. These differences are also reflected in the shell disorder of the respective viruses. 

### 5.2. HIV-1 and HIV-2: Similarities and Differences in Shell Disorder and Evolution

Differences in matrix disorder can be found among the different strains of HIV-1 and in HIV-1 and HIV-2. Although certain strains of HIV-1 could reach a PID of 70% and the mean matrix PID of HIV-1 is 56.5 ± 10.8%, some HIV strains have lower PIDs. The difference between HIV-2 (PID: 51.5 ± 2.5) and HIV-1 (PID: 56.5 ± 10.8) mean PIDs provides us with not just an explanation indicative of the differences between the two viruses but perhaps also explanatory hints pertaining to the differences among the various HIV-1 strains. HIV-2 is predominantly found in West Africa, where its reservoir lies in the population of the SIV-infected sooty mangabey monkeys that dwell in the rainforests [11,12,52]. Unlike the globally widespread HIV-1, HIV-2 therefore needs to be constantly replenished by the transfer of virus between the monkeys and humans via bites and consumptions of bushmeat. While both HIV-1 and HIV-2 are mainly sexually transmitted, HIV-2 has obviously a slightly greater non-sexual transmission component that provides for greater fitness if the virus is able to stay longer in the environment and in the primates’ saliva, which could potentially harm the virus. This explains the somewhat lower mean HIV-2 matrix PID (51.5 ± 2.5) that is not anywhere near the 60–70% seen in some HIV-1 strains. 

An interesting trend, that can be seen in not just HIV but also in viruses in general, is the tendency for a virus with a more disordered outer shell to have a more ordered inner shell and vice-versa. This can be explained if we see the virus as compensating the cost of having a soft outer shell by having a harder inner shell in an effort to protect its genome from the environment. Conversely, if the virus has a harder outer shell, it then has the luxury of more disordered inner shell proteins. This may explain why HIV-1’s innermost shell proteins (p7) drop dramatically in PID (39.5 ± 3.0%) especially when compared to HIV-2 (46.5 ± 0.1%). Oddly, the intermediate shell (capsid) PID of HIV-2 (26.6 ± 2.9%) is even lower than that of HIV-1 (44.5 ± 1.6%). Perhaps HIV-2 needs further reinforcement for its outermost shell (matrix) as a result of the harsher environment required in its spread via monkey bites and bushmeat consumptions. Another interesting note, given the differences in matrix PID of HIV-1 and HIV2, is the fact that HIV-1 is much more virulent than its HIV-2. HIV-1 kills over 90% of those infected within a few years of infection in the absence of antiviral drugs, whereas HIV-2 takes a much longer time to kill its host if at all [11,12,55]. The reason for this is not entirely clear but HIV-1 infections usually involve higher viral-load in host body. Apparently, the highly disordered matrix helps evade the host immune system. Another possibility is that the higher disordered matrix may allow greater promiscuous binding of host cells and thereby help increase the viral load [11,12]. 

### 5.3. HCV and HIV: Similarities and Differences in Shell Disorder and Evolution

While we have seen that HCV, like HIV-1, has a disordered outer shell, HCV outer shell PIDs do not reach the 60–70% PID levels seen in some HIV strains. One reason for the difference has already been mentioned. HCV is not easily transmitted by sexual intercourse but is seen as transmitted mainly by blood contamination especially via illicit drugs use. Interestingly, blood contamination does not usually require the virus to remain in harsh non-physiological environment considering the fact that the virus is likely to remain in an aqueous blood environment throughout the transmission. Incidentally, HIV-1 is also commonly spread among drug users via blood contamination [11,55]. A better explanation for the lower outer shell PID in HCV (52.5 ± 0.5, 48.5 ± 0.5), in contrast to HIV (56.5 ± 10.8), has to do with the fact that HCV is a hepatitis virus that is likely to be in contact at various times with bile, which is produced by the liver. Bile digests lipids and is known to be capable of damaging microbes such as bacteria [56]. 

### 5.4. HSV-1, HSV-2 and HIV: Similarities and Differences in Shell Disorder and Evolution

We have seen that HSV is a much more complex virus than HIV. This is true even with respect to its shell disorder and the transmission evolution. For example, it is difficult to thoroughly analyze the huge number of tegument proteins as it involves over 20 proteins [42]. The best we can do is to limit our study to just the major tegument proteins. We have also seen that modes of transmission of HSV are highly complex with HSV-1 and HSV-2 having different modes of transmission. While HSV-1 and HSV-2 are usually casually and sexually transmitted respectively, their evolutions and genetics are often intertwined as they are closely related. For instance, it was believed that genital herpes was solely the result of HSV-2 infections but nowadays with the practice of oral sex, clinical studies have seen cases of genital herpes arising from HSV-1 infections [57]. As if it is not already complicated, HSV-1/2 chimeras, i.e., a virus with both HSV-1 and HSV-2 genes, have been found worldwide [58]. Such is to be expected as it has been previously observed that in other viruses such as HIV, closely related viruses do often exchange genes regularly especially in cases when two viruses infect a host simultaneously [55]. 

Our data tell us that some of the disorder characteristics of HIV can also be found in HSV with, of course, differences. As mentioned, just as a highly disordered matrix can be found in HIV-1, high disorder can be found in the major tegument (matrix) proteins of HSV. We need, however, to remember that the minor proteins of HSV have not been considered and many of the minor (less abundant) proteins are ordered. Given the transmission mode by salivary contacts in HSV-1 and the constant gene exchanges between HSV-1 and HSV-1, some levels of order should be expected, unlike HIV. Differences in the tegument PIDs between HSV-1 and HSV-2 are also found to be in the right “direction” i.e., the more sexual HSV-2 being more disordered than the more casually transmitted HSV-1. As seen in Table 1, HSV-2 has three major tegument proteins (UL49, UL47, UL48) that have higher PIDs (2–3% higher) than the corresponding proteins of HSV-1. HSV-1, on the other hand, has only one protein than is higher than that of HSV-2 but that is higher by just 0.5% (UL36) in contrast to the 2–3% seen in the three mentioned proteins. It is also misleading to assume that the percentage differences are insignificant because they are small. The proteins involved are large and therefore the number of residues involved can be relatively substantial. For example, UL47 is 693 residues long. Furthermore, it is also possible that the disordered regions are strategically located in closer proximity to an outer membrane where it matters most but we do not know this for sure. Lastly, we should not forget that the proteins mentioned are major tegument proteins. In other words, copies of the proteins are abundantly found in the tegument [42,53].

## 6. More than One Mode of Evasion: Latency 

Although the absence of effective vaccines and the presence of a relatively disordered outer shell represents the common traits, they are, by no means, the only common characteristics defining virulence. In fact, one should also take into account the uncanny ability of viruses to hide in latency [2,5]. In our view, the promiscuity of the disordered proteins or proteins with disordered regions is likely to play a role in infecting a greater number of cells where the virus can hide in [11,30]. 

## 7. Experimental and Empirical Evidence Has Trickled in During the Last 10 Years

As mentioned, we first reported the particularity of HIV matrix and made the connection to the ability of this virus to evade the host immune system in 2008. Ten years have since passed and some experimental and empirical evidence has been accumulated. As already mentioned, a shell disorder database of over 300 viruses and strains including some of the most infamous and deadly viruses have been collected and analyzed. The results of these analyses have been very consistent with respect to what is already known about viruses, including vaccine availability and their modes of transmission. Much, not all, of the results have already been documented and published [3,11,12,17,18,19,20,22,25,26]. The results are simply too numerous to be thoroughly examined in this paper.

Nuclear magnetic resonance (NMR) studies have re-affirmed the presence of long and short disordered regions in the p17 of HIV [59]. Furthermore, several studies have shown that the HIV matrix has an ability to induce B-cell growth [60,61]. One particular study has shown that when p17 becomes more disordered as a result of a point mutation, it loses its ability to elicit B-cell growth, even though the precise binding sites responsible for such elicitation are unknown [61]. Such experiments are important as they prove that the HIV matrix can affect interactions of antibodies with HIV, thereby providing support to the viral shapeshifting paradigm. Experimental evidence is also accumulated to show that the rate of evolution of viruses is correlated with their modes of transmission. For example, it has been shown that HSV that has been paternally transmitted is genetically different from those transmitted via other modes [62]. Computational and empirical models using methodology similar to the already described one have been able to establish the relationships between the levels of shell disorder and modes of transmission among coronaviruses. The model was so accurate that it was able to predict that MERS-CoV has a strong fecal-oral transmission component and therefore would not spread as easily as its cousin, SARS-CoV [20,22]. These studies showed the important roles that shell disorder plays in the evolution of viral transmission, which gives additional credence to viral shapeshifting theory. In yet another study, high positive correlation between Ebola nucleocapsid disorder and virulence was seen [25]. Similar correlations could be found between flavivirus capsid and virulence [26]. Since these studies involve inner shells of the related viruses, they provide further validation for the existence of viral shapeshifters of a different kind contrasting the HIV, which has a disordered outer shell and is a shapeshifter of a unique kind. 

A recent review of past literature has led to the revelation that the mechanisms involved in the here described correlation between the intrinsic disorder levels and virulence of the viral shells had actually been unknowingly discovered in the 1920s by the legendary scientist, Oswald Avery [13,14,15]**.**

### Mechanism of Immune Evasion for the True Viral Shapeshifter

At the end of the 1920s to the early 1930s, Oswald T. Avery and Walther F. Goebel conducted a series of classical experiments that have greatly contributed to the successful design of many vaccines even today [13,14,15]. In that studies, they synthesized polysaccharides of bacteria Pneumococcus and inoculated the respective samples into rabbits. However, they found out, much to their dismay and puzzlement, that there was no immune response. They, however, came up with an idea of tying the polysaccharides together using a protein linker. Then, they injected the glyco-conjugate, as it was called, into the rabbits, and this time they observed expected immune response [13,14,15]. Interestingly, it was also found that the closer the linker resembles the natural bacterial protein linker, the greater the immune response was. These important observations are in line with our hypothesis, since we know now that many bacterial proteins are well-ordered, especially proteins found in bacterial shells, as bacteria often have to dwell in harsh places [63,64]. The two facts help reinforce the idea that the best immune response can be derived when the polysaccharides are held tightly together via rigid linkers. A corollary to this knowledge is that the more flexible the linkers are, the likelier it is the immune response will be poor. This corollary forms the experimental basis of the concept of the viral shapeshifting or shell disorder and immune evasion.

**Figure 4:**
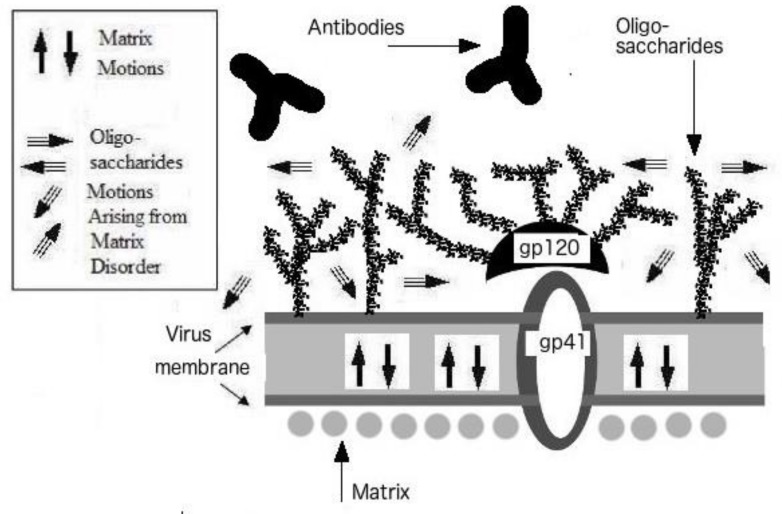
Mechanism of Immune Escape. Figure reproduced with the permission of Gerard KM Goh, 2017.

Figure 4 illustrates how the aforementioned corollary applies to our case. The true viral shapeshifter has a highly disordered outer shell, that is engaged in constant motions. These motions will then enhance the motions of the surface glycoproteins and thereby prevent their tight binding to antibodies. It is little wonder that while antibodies that bind to HIV are easily found, it has been virtually impossible to find neutralizing antibodies for this virus [65]. The lack of the neutralizing antibodies themselves presented a huge mystery that had been nagging the biomedical community until now. Yet another piece of evidence supporting these mechanisms is the fact that HIV is one of the most glycosylated viruses known-to-date [66].

## 8. Implications and Potential Applications of the Viral Shapeshifters: Cancer and Bacterial Virotherapy as an Example

Obviously, researchers involved in the HIV vaccine development need to go through the shell disorder data using the viral shapeshifting concepts presented here to determine the feasibility of their strategies and to perhaps devise new strategies. To improve efficiency in vaccine discovery, potential vaccine candidates could be best screened against HIV-1 with the highest matrix disorder as different strains of HIV-1 have various levels of matrix disorder. Furthermore, since viral shell disorder has been associated with virulence and modes of transmission [11,12,20,22,25,26], shell disorder data therefore could become a useful tool for vaccine developers and epidemiologists. 

An even more intriguing potential area of application is virotherapy against bacterial infections or cancer. Viruses that infect bacteria are known as phages or bacteriophages. They were first discovered in the early 20th century by Frederick Twort and Felix d’Herelle [3,4,67]. With the rise of the antibiotic-resistant bacteria, phage therapy is becoming a more viable alternative treatment for bacterial infections [68]. Similarly, advances in cancer treatments have allowed the cancer virotherapy to gain greater spotlight [69,70]. Viruses do have oncolytic potentials or abilities to lyse cancer cells, because they share the same signaling systems, and cancer cells often over-express receptors necessary for viral entry [71]. The oncolytic herpes virus, T-Vec, has been approved by the Food and Drug Administration [72]. 

A major obstacle for both phage and oncolytic virotherapies is the accurate delivery of the viruses to the target. One factor is that the host immune system will attempt to neutralize the therapeutic virus even before it reaches its target and thereby will render the viral agent ineffective [68]. This is where the concept of viral shapeshifting will come in handy. Viruses can be engineered to behave like HIV-1, which, as we have seen evades the immune system efficiently and moves easily to organs that are normally difficult to reach, like the brain [54]. Another factor is that cancer cells can hide out in poorly accessible places, such as the brain. Here again, the concept of shell disorder can come in handy, as protein intrinsic disorder allows for greater binding promiscuity [30] and therefore can help viruses to enter into vital organs, such as the brain [12]. In line with these considerations is the fact that HIV enters the brain and other vital organs with ease.

Additional experiments on bacteria provide further support for the hypothesis presented herein, namely that highly flexible proteins and regions and (e.g., IDPs and IDRs) frequently make poor antigens. The fibronectin binding protein (FBP), which is anchored onto the surface of *Staphloccoccus Aureus*, has been shown experimentally to be entirely disordered except for its membrane anchor region [73,74], and, accordingly, the IDR extension is predicted to be so by PONDR VL-XT (unpublished). As is commonly observed for IDRs, FBP undergoes a disorder-to order transition as it binds tightly to its partner, fibronectin [75]. While in principle antibodies against FBP would be an effective therapy against *S. aureus*, attempts to raise such antibodies have not been successful [74]. Similar failures to obtain antibodies against the host-targeting proteins have been observed for several pathogens not counting the three viral examples discussed herein, suggesting that the evolution and utilization of disorder-to-order transitions for binding to host targets may be a strategy commonly used by pathogens to avoid immunological neutralization [27,76]. 

## Figures and Tables

**Table 1:** Viruses with sample UniProt accession codes for shell proteins.

Virus	Virus Type, Tansmission	Outer Shell, Proteins (UniProt Accession)^+^	Intermediate Shell	Inner Shell
EIAV	*Retroviridae* (RNA) Insect	Matrix, p15 (P69732)	Capsid, p26 (P69732)	Nucleocapsid, p11 (P69732)
Influenza	*Orthomyxo-viridae* (RNA) Resspiratory	Matrix, m1 (P05755)		Nucleoprotein NP (P21433)
HIV-1	*Retroviridae* (RNA) Sexual	Matrix, p17 (P03348)	Capsid, p24 (P03348)	Nucleocapsid, p7 (P04594)
HIV-2	*Retroviridae* (RNA) Sexual, Bite	Matrix, p17 (P04584)	Capsid, p24 (P04584)	Nucleocapsid, p7 (P04584)
Variola/Smallpox*	*Poxviridae* (DNA) Inhalation	Membrane, C9L (Q76U97), A14 (P33839), F5 (P33865)		Core, VP8 (Q0N570), 4A (Q0N532), 4B (Q0N539)
Rabies	*Rhabdoviridae* (RNA) Bites	Matrix, M (P25224)		Nucleocapsid, N (P151979)
Poliovirus	*Picornaviridae* (RNA) Fecal-Oral		Capsid, VP1-4 (P03302)	
Yellow Fever (YFV)	*Flaviviridae* (RNA) Insect	Membrane, M (P03314)	Capsid, C (P03314)	
Hepatitis C (HCV)	*Flaviviridae* (RNA) Sexual, Blood Contamination			Core, p19, p21 (P26663)
Herpes Simplex Virus-2 (HSV-2)*	*Herpesviridae* (DNA) Oral-Oral Contacts	Tegument, VP22-UL49 (D3YPK7), VP1/2-UL36 (I1UYK0), VP13/14-UL47 (P10231), VP16-UL48 (P06492)		Capsid, VP5 (P06491)
Herpes Simplex Virus-2 (HSV-2)*	*Herpesviridae* (DNA) Sexual	Tegument, VP22-UL49 (A7LK33), VP1/2-UL36 (G9I258), VP13/14-UL47 (A7LK25), VP16-UL48 (P68335)		Capsid, VP5 (P89442)

* Only major shell proteins are considered.^+^UNIPROT: http://www.uniprot.org.

**Table 2:** Percentage of Intrinsic disorder (PID) levels of shell proteins.

Virus	PID (%) of Outer Shell	PID (%) of Intermediate Shell	PID (%) of Inner Shell	Vaccine Available ^+^
EIAV	13 ± 0.1	29 ± 0.1	26 ± 0.1	Yes
HIV-1, SIVcpz ^#^	56.5 ± 10.8	44.5 ± 2.6	39.5 ± 3.0	No
HIV-2, SIVmac ^+^	51.5 ± 2.5	26.6 ± 2.9	46.5 ± 0.1	No
Influenza	35.2 ± 1.6		44.7 ± 5.4	Yes
Smallpox	13 ± 0.1		19 ± 0.1	Yes
	8 ± 0.1		4 ± 0.1	
	15 ± 0.1		12 ± 0.1	
Rabies	25.8 ± 1.4	21.5 ± 0.8		Yes
Poliovirus		34 ± 3.8		Yes
		15.12 ± 6.1		
		31.3 ± 3.6		
		27 ± 0.1		
Yellow Fever (YFV)	35.2 ± 0.9		74.3 ± 0.9	Yes
HCV			52.5 ± 0.5 48.5 ± 0.5	No
HSV-1	58.0 ± 1.4		18 ± 0.1	No ^
	50.5 ± 0.5			
	36.3 ± 0.5			
	37 ± 0.7			
HSV-2	61 ± 1.6		18 ± 0.1	No
	50 ± 0.1			
	38 ± 1.1			
	39.3 ± 0.1			

* The standard error is denoted by the prefix “±”. PIDs are arranged according to proteins as stated in Table 1. For example, the HCV core PIDs 52.5 ± 0.5 and 48.5 ± 0.5 refer to core proteins p19 and p20 respectively as arranged in Table 1. ^#^ Simian Immunodeficiency Syndrome—Chimpanzee (SIVcpz), ^+^ Simian Immunodeficiency Syndrome—Macaque (SIVmac). ^^^ A somewhat effective potential vaccine has been detected for HSV-1 during clinical trial. The further clinical trial for this vaccine candidate has been discontinued indefinitely because of the complete failure in the HSV-2 part of the same trial.
